# The “goldfish bowl”: a qualitative study of the effects of heightened surveillance on people who use drugs in a rural and coastal Canadian setting

**DOI:** 10.1186/s12954-022-00725-2

**Published:** 2022-12-07

**Authors:** Geoff Bardwell, Manal Mansoor, Ashley Van Zwietering, Ellery Cleveland, Dan Snell, Thomas Kerr

**Affiliations:** 1 School of Public Health Sciences, University of Waterloo, 200 University Avenue West, Waterloo, ON N2L 3G1 Canada; 2 British Columbia Centre on Substance Use, 400-1045 Howe Street, Vancouver, BC V6Z 2A9 Canada; 3 Department of Medicine, University of British Columbia, St. Paul’s Hospital, 608-1081 Burrard Street, Vancouver, BC V6Z 1Y6 Canada; 4 qathet Community Action Team, 218-6975 Alberni Street, Powell River, BC V8A 2B8 Canada; 5 Lift Community Services of qathet Society, 218-6975 Alberni Street, Powell River, BC V8A 2B8 Canada; 6 Substance Users Society Teaching Advocacy Instead of Neglect (SUSTAIN), 218-6975 Alberni Street, Powell River, BC V8A 2B8 Canada

**Keywords:** Rural Canada, People who use drugs, Goldfish bowl effect, Surveillance, Stigma, Criminalization

## Abstract

**Background:**

A growing body of research has focused on contextual factors that shape health and well-being of people who use drugs (PWUD). However, most of this research focuses on large cities and less is known about the effects of social and structural contexts on drug use and associated risks in rural Canadian settings. Therefore, we undertook this study to examine rural-specific contextual factors that affect the day-to-day experiences of PWUD.

**Methods:**

Twenty-seven qualitative semi-structured interviews were conducted with PWUD in a rural and coastal setting in British Columbia, Canada. Participants had to be ≥ 19 years old, used illegal opioids and/or stimulants regularly, and lived in the qathet region. Interview transcripts were coded based on themes identified by the research team.

**Results:**

Participants described progressive shifts in politics and culture in the qathet region while also identifying resource scarcity, homelessness, and changes in the drug supply, where illicit drug contents have become highly toxic and unpredictable. Participants discussed the qualities of a small community where everyone knows each other and there is a lack of privacy and confidentiality around drug use, which resulted in experiences of stigma, discrimination, and surveillance. Participants also reported rural-specific policing issues and experiences of surveillance on ferries when traveling to larger cities to purchase drugs. This led to significantly higher drug prices for PWUD due to the time dedication and criminalized risks associated with drug possession and trafficking.

**Conclusions:**

Our findings illustrate the unique experiences faced by PWUD in a rural and coastal setting. The “goldfish bowl” effect in this rural community created heightened social and structural surveillance of PWUD, which led to a variety of negative consequences. There is a clear need for interventions to address the larger contextual drivers affecting people who use drugs in rural settings, including decriminalization and peer-led anti-stigma strategies, in order to improve the lives of PWUD.

## Introduction

### Background

There is a growing body of the literature that documents the impact of a range of contextual factors on people who use drugs (PWUD), drug use-related risks and harms, as well as how these factors affect the implementation of targeted harm reduction interventions such as needle and syringe programs and supervised consumption sites [1–3]. For example, multiple studies have examined how the built environment (e.g., neighborhood characteristics, health service layout and design, sanitary conditions) impact drug use and related risks and harms [4–8] as well as service access and delivery [9–12]. Studies have also focused on the effects of social factors (e.g., gender norms, stigma, discrimination) on the experiences of PWUD [13–21].

Given the ongoing criminalization of illicit drugs, researchers have also examined the surveillance and policing of PWUD [8, 22–33]. For example, in their qualitative study on injection drug use in three Russian cities, Sarang et al. [29] describe how policing practices, including surveillance, extortion, and physical violence created fear, internalized stigma, and social suffering among PWUD. While importantly illuminating the effects of criminalization and surveillance of PWUD, the overwhelming majority of this research has been conducted in large urban settings. There is a paucity of research that has explored contextual factors affecting the experiences of PWUD in rural settings [34, 35], and existing studies tend to focus on issues related to accessing treatment, healthcare, and harm reduction services and primarily focus on rural settings in the USA [36–45] and therefore may not be applicable to the Canadian context given the differences in drug policies, healthcare access, and novel harm reduction approaches. There is also a dearth of qualitative studies on rural-specific experiences of PWUD in a Canadian context [35]. Therefore, this study aims to characterize the contextual factors that shape drug use and the experiences of PWUD in a rural and coastal community in British Columbia (BC), Canada.

### Study setting

The qathet region of BC—the setting of this study—has a population of approximately 21,000 people and covers 5000 square kilometers of land [46]. It is on the traditional territories of the Tla’amin, Klahoose, Homalco, and Shíshálh People and includes several communities on the mainland (e.g., Powell River, Lund, Tla’amin Nation) and coastal islands with varied levels of accessibility by ferry, water taxi, or private charter (e.g., Texada Island, Savary Island, Lasqueti Island). The economy in the region is primarily driven by blue-collar industries, including forestry and a local paper mill, as well as recreation and tourism. However, the former has been negatively impacted by the closure of the paper mill [47] and latter has been greatly affected by the ongoing COVID-19 pandemic due to travel restrictions and public health orders. The region is approximately 175 km north of Vancouver, BC, and is only accessible by road transportation and two ferry crossings or air travel via a small airplane. The region has been significantly impacted by the ongoing overdose epidemic, and in 2021, it had a mortality rate that was more than double the provincial average [48]. A variety of harm reduction programs and services exist in the region, including an overdose prevention site, an injectable opioid agonist therapy clinic, a peer-led advocacy group (i.e., Substance Users Society Teaching Advocacy Instead of Neglect), and a naloxone training and distribution program [49]. However, to date, no research has been conducted examining the experiences of PWUD in this rural and coastal region. This article focuses on the experiences and perceptions of PWUD in the qathet region and how social and structural contexts in a rural setting shape their day-to-day lives.

## Methods

Data collection occurred between July and October 2021. Due to pandemic-related restrictions, remote qualitative one-to-one interviews were conducted over the phone from the home offices of the study team and participants were located either in their homes or in a private office at a local drop-in space in Powell River. To be eligible to participate in an interview, participants were required to be over the age of 18; regularly use illicit opioids and/or stimulants (i.e., 3–4 times per week or daily); and live in Powell River or the surrounding qathet region. Recruitment posters were placed in common areas of several local health and social service providers, which were in full operation at time of data collection. Posters were also distributed through community partners. Posters directed potential participants to speak with our research collaborators and co-authors (AVZ and EC) who either worked in harm reduction and clinical settings and/or frequently engaged with potential participants on a regular basis (including in locations outside of clinical settings such as supportive housing). Eligibility screening was conducted by AVZ and EC who then provided study information and consent forms to participants and scheduled phone interviews with those who were eligible and who provided written informed consent.

We consulted with local PWUD, clinicians, and other community stakeholders, to develop our research objectives and interview guide questions. The overall study aims were to characterize the unique features of the qathet region as they related to drug use and health and well-being; to examine the impact of social, structural, and environmental contexts on the day-to-day lives of PWUD; and to understand the impact of contextual factors on access to, and uptake of, evidence-based drug use services. This article focuses on data related to the first two aims, with subsequent articles exploring the third aim.

We purposively sampled 27 participants to participate in this study, and we utilized a demographic checklist to ensure a diverse representation. For example, after 15 participants were interviewed, we realized that the majority lived in supportive housing or were more centrally located and so we refocused our recruitment to target those who live in other communities across the qathet, including people living on coastal islands. Interviews were conducted remotely and audio recorded by GB and MM and the average length of time per interview was approximately 45 min to one hour. Cash honoraria ($30 CAD) was distributed to participants by AVZ and EC.

Interviews were transcribed verbatim by a professional transcription service and checked for accuracy by the lead author. GB and MM reviewed a selection of transcripts as well as any interview notes to develop a coding framework based on themes identified by the research team [50, 51]. NVivo 12 was utilized to organize and code the dataset based on our coding framework [52]. Preliminary findings were presented to a group of community stakeholders (i.e., qathet Community Action Team) to gain feedback, strengthen the validity of our results, and prioritize topics for publication and other knowledge translation activities. This study was approved by the Providence Health Care/University of British Columbia Research Ethics Board. Due to the small size of the community in the qathet region, and based on feedback from our community partners, we decided to not include demographic information after each quotation to protect participant confidentiality and anonymity. See Table 1 for participant characteristics.

**Table 1:** Participant characteristics (n = 27)

*Age*	
Range	19–65
Average (mean)	42
*Gender*	
Ciswoman	13
Cisman	14
*Race*	
Indigenous	8
White	19
*Housing*	
Supportive housing	13
Private market housing	6
Shelter	4
Unsheltered	4
*Drug use (last 30 days)*	
Fentanyl	25
Crystal methamphetamine	17
Cocaine (rock)	14
Heroin	12
Cocaine (powder)	7
Cannabis	6
Alcohol	6
Other	6
*Consumption method*	
Smoke	24
Inject	18
Swallow	5
Snort	1
*Income (last 30 days)*	
Social assistance	26
Part-time job	8
Drug selling	6
Gig economy	6
Full-time job	2
Selling goods	2
Volunteer stipend	2
Sex work	1
Recycling	1
Panhandling	1

## Results

Of the total 27 participants, the most common drug consumption method was smoking and fentanyl was the drug that was used most. Nineteen participants had housing (either supportive or private market), and eight were experiencing homelessness. The majority of participants received income assistance (n = 26). See Table 1 for further participant characteristics. To help explain the study participants’ experiences of surveillance in a rural and coastal community, we utilize the analogy of the “goldfish bowl” effect, which demonstrates how a goldfish can be viewed from all directions and how the bowl magnifies the goldfish.

### Changing coastal contexts

Most of the participants lived in Powell River (or close-by), which is the largest community in the qathet region with a population of approximately 14,000 people. When we asked participants to tell us about where they lived, most described the remoteness and the vast geographical area in and around Powell River with an emphasis on the scenery and outdoor recreational activities. Aside from the natural elements, participants also described their experience growing up in the area, including cultural shifts. Powell River was described as politically progressive in some regards with a strong arts community in recent years. For example:

*It has almost always been an NDP [i.e., New Democratic Party; a left-of-centre provincial party] town, politically. We have a gay MLA [Member of Legislative Assemble] and a former cocaine dealer as a mayor. So, it’s pretty progressive and it’s very arts-focused…It was a rather redneck forestry logging town when I was growing up, but I guess right after high school, when the [paper] mill was winding down quite a bit, there must have been some kind of a shift, and the arts really took over and kind of redefined the town.* (P10)

Despite some describing these progressive cultural shifts, when asked to describe some challenges with living in Powell River, all participants were quick to describe a lack of resources, employment opportunities, and housing options, particularly for PWUD and those living in poverty. Heightened housing costs resulting in more homelessness were described as a newer challenge in the region, which may, in part, be a result of migration from larger cities in BC and the lack of affordable housing options for people on income assistance programs [53]. According to one participant, *“Like there were no homeless people in this town my entire life here until like five years ago. And then all of a sudden there’s 50 of us plus living on the street”* (P22). Further, participants described illicit drug use as *“rampant”* and *“worse than bigger cities, drug scene-wise”* (P9) and they discussed shifts in illicit drug markets, with higher toxicity and unpredictable contents. For example: *“The drug supply has become almost unbearably toxic here…we’ve gone from heroin to fentanyl cut with heroin to just straight benzodiazepines cut with caffeine”* (P1).

### Social contexts: “There is a lot of judgement”

Participants described Powell River as an isolated and tight-knit community. According to one participant, *“It’s just more cut off I’d say it feels like. There’s definitely very little resources here. But it’s just like it’s like this regular small town. You know, everyone knows each other kind of thing”* (P22). Participants uniformly described the social challenges of living in a rural community where *“everyone knows everybody”* (P25) and *“everyone knows what’s going on in this town”* (P7; goldfish bowl effect). For example:

*Small town people talk, talk, talk. The rumours fly around like crazy. That’s what it’s like in a small community. That’s how it’s always been growing up…People blow shit out of proportion because they’re bored and they got nothing better to do than talk about other people.* (P23)

Participants reported that this is especially challenging for PWUD as they described a lack of privacy and confidentiality regarding their drug use, similar to the goldfish bowl effect and being viewed from all directions. For example:

*They call it ‘Powell Rumour’ because it’s actually so bad here for that kind of stuff. Like the walls have ears, I swear…stuff travels in this town like you wouldn’t believe. Like I had an overdose one time and it actually made it to my family from a [healthcare worker].* (P6)

*With that small of a town, it’s pretty hard to have any kind of confidentiality, right? Depends on where people see you, right? For instance, I used to go hang out at my buddy’s house and they sold drugs at the house, so therefore [people] are like ‘Oh, you’ve got to be up to something,’ right? Which is very shitty…At the time, I wasn’t actually even doing drugs.* (P18)

Participants not only described a lack of privacy and confidentiality, but also the consequences it had for PWUD in rural settings, including stigma, discrimination, and surveillance as evident in the following quotes:

*A lot of people do judge, I’ve noticed especially in Powell River they do judge. There are a lot of people who look at you differently if they know you use [drugs].* (P12)

*This town, the entire town knows who I am. There’s no privacy in a small town. I go into a store, and I’m asked to leave because I’m a drug dealer. Or I get dirty looks all throughout the town. It’s because of my addiction that they are judging me.* (P5)

Multiple participants also described similar experiences when accessing their opioid agonist therapy medications (e.g., methadone) from a local pharmacy feeling *“uncomfortable”* and *“judged.”* For example:

*I go to [the pharmacy] and you’re on your own little booth side, right? So, anyone that comes for a prescription is on one side and anyone that comes for narcotics is on the other side. I feel that we should all be in the same area. I’m getting a prescription and this person is getting a prescription but they’re picking it up there, and I got to pick mine up over here. It’s like being in jail.* (P18)

As a consequence of their negative daily experiences across multiple settings, some participants reported avoiding public settings at all costs as a way to maintain privacy where *“prying eyes aren’t watching”* (P17). For example:

*I try and just limit my outings. I go from point A to point B. I don’t stick around trying to look for trouble. Nobody chooses when they wake up to be an addict and yet people are so fast to stigmatize them.* (P9)

Surveillance of PWUD was reported as commonplace in Powell River, and participants described these experiences as heightened in a rural setting given the smaller population size, stigma toward PWUD, and a culture of hearsay. Taken together, these produce a goldfish bowl effect for PWUD in a rural setting.

### Structural contexts: policing, drug costs, and “the gauntlet”

The heightened surveillance in Powell River was not just limited to social contexts and their effects on PWUD. Participants also reported negative experiences of surveillance as they related to law enforcement and criminalization, amplifying the goldfish bowl effect beyond the social realm. Some participants described police violence and harassment of PWUD as evident in the following quote:

*Our cops over here, you treat them with respect, they don’t treat you with respect, they treat you like a fuckin’ criminal even if you’ve never done nothing wrong. They’ll throw you to the ground and like beat people. Like I’ve been beat by the cops…It’s a small town problem for sure.* (P27)

For participants who had experience living in other larger cities, they described differences in policing approaches in a rural setting, partly because cities tend to have their own police forces whereas smaller communities rely on federal policing (i.e., Royal Canadian Mounted Police [RCMP]). Police in rural settings were reported as being less lenient and more stern regarding enforcement. For example:

*City police are way different than RCMP rookies. Yeah, the old staff sergeant retired last year so we’ve got a new one and the RCMP up here are pricks actually. There’s a huge difference…They’re assholes up here.* (P8)

Participants described the routine police profiling and surveillance experienced among PWUD in the qathet region and how there are differences between rural policing and city policing: *“In Vancouver your dope would be tossed. Here, you’re charged, your life’s destroyed basically”* (P3).

Criminalization and surveillance were discussed as having direct financial implications for PWUD. All participants described how drugs cost significantly more (e.g., 300–400% more) in Powell River compared to cities like Vancouver and Victoria. For example: *“I don’t pay more than 30 bucks now for a point and sometimes I can get it cheaper, but compared to Vancouver it’s still pretty pricey”* (P4). Some participants were able to acquire heroin that was not cut with fentanyl, but this also came at a steep cost: *“It’s really costly and not very many people can get it. I can get it but it’s expensive. Really expensive. I would have to pay double. A half a gram would cost me about $200”* (P8). When asked about why drugs costs significantly more than in bigger cities, participants discussed the far distances people have to travel to acquire drugs: *“it’s harder to get them here I guess”* (P25). Travel to Vancouver, for example, was described as requiring a car (or an infrequently scheduled bus system) and involves two ferry crossings and many hours of travel time.

Multiple participants described being closely watched by others when traveling to the city to *“score”* or *“pick up.”* For example:

*There is a bus, there’s a transit system here. It’s a barrier to go pick up, it’s a nightmare if you’re really gonna pick up in a smart way and pick up bulk because you need to go down to Vancouver or wherever, Victoria, wherever people pick up…we call the ferries the gauntlet because the ferry workers think they’re frickin’ border security and they’ll call the cops on anybody.* (P3)

During the time of data collection, there were pandemic-related travel restrictions across the province, which created additional barriers for people wanting to score drugs in the city. According to one participant,

*Well there’s no travel on the ferries. You got to show your medical passes pretty much [i.e., travel exemption]. The cops are patrolling the fucking ferry terminals…See who’s driving what and who’s going where, why. You got to roll down your window and tell them why you’re getting on the ferry. Fucking assholes. ‘Going over to get a blow job,’ I tell them. That’s all I tell them. They don’t even look at me anymore, thank God.* (P8)

Travel restrictions coupled with drug criminalization and a culture of surveillance in a rural setting (i.e., the magnification of the goldfish bowl) were described as creating a lot of barriers and risk for PWUD. For example:

*Like with scoring, scoring is extremely stressful…and depending on what you’re doing you have to be very careful. You need a lot of money to really score and make it worth your while if you’re gonna risk, risk drug dealing and risk your life charges, it’s unsafe…I don’t want to be in a crack shack scoring and I don’t trust people…and, it’s just like a waste of money if you get ripped off. Travelling is expensive. It’s always nice to get out of Powell River anyway but I, I think I’d rather go to a concert or something.* (P3)

*You have to bring it on the boat, which is high risk too. So, people compensate themselves for their trouble.* (P10)

Unsurprisingly, the risks and challenges associated with traveling to purchase drugs from the city and traveling back to the qathet region resulted in significantly higher costs, which many reported as unaffordable and consequently led to the need to *“do crime”* to support their drug use.

## Discussion

In summary, while some participants described progressive cultural shifts in the qathet region, most identified a variety of negative experiences including those related to their drug use. Participants discussed a lack of privacy and confidentiality around drug use in a rural setting, which resulted in experiences of stigma, discrimination, and surveillance. Participants also reported rural-specific policing issues and experiences of surveillance on ferries when traveling to larger cities to purchase drugs, leading to higher drug prices due to both the time dedication required and criminalized risks. Taken together, these findings denote rural-specific social and structural contexts, the implications that they have for the lives of PWUD, and the need for policy and practice interventions to address the negative effects of socio-structural surveillance.

The day-to-day experiences of study participants were impacted by a “goldfish bowl” effect, which illustrates the visibility of the goldfish from all directions and how the glass bowl magnifies the goldfish. This analogy is highly applicable in understanding the experiences of PWUD in a rural setting, where participants reported a culture of surveillance and a lack of privacy and confidentiality regarding their personal lives, including their drug use. This pervasive culture is also similar to what Foucault refers to as a carceral society whereby the carceral system extends to a society that judges all and excludes those who fall outside of social norms [54]. The literature on the goldfish bowl in rural settings primarily focuses on issues related to clinical practice and mental health service access. For example, some clinicians have described their personal hardships from a lack of anonymity due to being both a community member and a professional in smaller communities [55–57]. Others have described the challenges of the goldfish bowl specifically for people with mental health issues in rural settings [58, 59], which are similar to the experiences reported by our study participants. According to Slama,

*Despite the isolation involved in rural living, there is also what I shall call a goldfish bowl effect, in which [individuals] are aware that other people are very interested in their lives and in talking to others about them. This lack of anonymity or privacy results in certain conventional behavioral expectations as well as pressure to conform to them.* [59]

Slama also describes potential outcomes of this rural phenomenon, including the magnification of stigma around mental illness and individuals attempting to hide certain aspects about themselves [59]. Participants in our study identified stigma from others due to their drug use, and as a result, some avoided being seen in public due to their negative experiences in the goldfish bowl.

While not describing the goldfish bowl specifically, there is minimal qualitative research that has examined the social effects of stigma more generally on drug use in rural settings. Similar to our findings, Ezell et al. have described how rumors about, and angst toward, PWUD have led to social stigma that is harder to escape in a smaller setting [42]. In their study on stigma and overdose risk in rural Kentucky, Fadanelli et al. describe how small communities are tightly connected which results in information circulating faster and heightened stigma compared to larger cities where PWUD have more anonymity [60]. Others have identified how stigma and a lack of confidentiality in rural settings affects access to health-related services for PWUD [61–63]. While importantly identifying the experiences of stigma among PWUD in rural settings, the majority of research in this area is based in the USA and focuses specifically on the social context of stigma.

We note that previous scholars have documented stigma in shaping the experiences of PWUD in rural settings [42, 60, 62]. We have sought to extend this literature by highlighting how structural forces converge with social processes like stigma to create additional harms via surveillance. Our findings demonstrate how the goldfish bowl and the stigma experienced by PWUD in rural settings are not just part of social contexts that shapes attitudes and behaviors, but they also permeate structural contexts (e.g., drug laws, policing, pharmacy policies). Participants from our study did not simply just experience stigma through rumors and gossip from others in the community; they also described the coinciding judgment and surveillance in pharmacy and public settings, which led some to limit their public outings. Pharmacy policies that require PWUD to retrieve their opioid agonist therapy medications from a distinct area demarcated a segregation that clearly identified them to others as PWUD. Studies on experiences of stigma in healthcare settings have shown how these can result in healthcare delays and avoidance leading to negative health outcomes [64–66]. Additionally, participants experienced heightened surveillance from police and security guards on ferries. There exists a plethora of qualitative studies examining the effects of surveillance and policing on PWUD [22, 29, 32, 67–70], but this almost exclusively focuses on drug use in large urban settings. In their qualitative comparative analysis on needle and syringe program access among PWUD in New York City versus rural Illinois, Ezell et al. report that rural PWUD chose drug use locations based on an ability to hide from the general public in contrast to urban PWUD who emphasized avoiding law enforcement [71]. This is not consistent with our findings, as participants reported experiences of surveillance from both the general public *and* from the police. This difference may, in part, have to do with rural policing in the Canadian context (i.e., the RCMP), and how our participants reported violence and harassment and a heightened surveillance and consequences from the RCMP specifically compared to their experiences with community police in larger Canadian cities (e.g., Vancouver). Given the small population size in rural communities where there is a lack of anonymity, police and ferry security guards are able to more easily identify local PWUD. Those who are criminalized become “marked” by stigma and this negatively affects their day-to-day experiences [72–74]. Studies have examined societal attitudes toward PWUD among professional stakeholders, including those of police, and research has reported negative perceptions where, for example, individuals are blamed for their behaviors or they are perceived as violent and erratic, and how these attitudes influence how PWUD are treated across various settings (e.g., zero-tolerance policies) [75–80]. However, additional research with police and security services in our study setting is needed to understand attitudes toward PWUD and how these perceptions might affect policing practices in rural Canada specifically. As our results demonstrate, the pervasive culture of surveillance has direct impacts on PWUD in rural settings not just in terms of their experiences of judgment and stigma from their community, but also in terms of their experiences of criminalization. Furthermore, this heightened surveillance creates risk for PWUD when traveling to the city to buy drugs in bulk, and as a result, significantly affected illicit drug prices.

Our study has implications for policy and practice in rural Canadian settings. It is evident that rural-specific drug use stigma and surveillance need to be addressed. There have been a variety of studies examining the impacts of stigma on PWUD as well as ways to combat it [15, 81–83]. Strategies tend to include education and awareness campaigns that present accurate information about drug use as well as literacy campaigns intended to improve knowledge and attitudes [83]. Contact-based anti-stigma interventions are another strategy (e.g., workshops or other interventions between PWUD and the general public), which are built on the premise that a lack of contact between groups fuels fear and discomfort and bringing different groups together would provide opportunities for connection [83]. However, whether these lead to attitude or behavioral changes across all settings remains inconclusive [84]. Peer-led anti-stigma strategies may be more successful in a smaller setting such as the qathet region, where some of the authors have direct experience making systems level changes—though evaluation efforts would be required to measure any outcomes related to anti-stigma strategies. Additionally, many PWUD in the qathet region have deep roots within the community so how this may be applied in a rural context is integral to consider. Thus, local organizations comprised of PWUD should be at the forefront of these strategies. To avoid placing additional responsibilities on an oppressed group, these anti-stigma strategies must also include meaningful employment for PWUD in order to improve their economic conditions and avoid precarious labor often placed on affected communities [85]. Importantly, any targeted strategies must work to address not only stigma at the individual and community level but also at larger structural levels, including surveillance, as addressing stigma in a meaningful way also requires alleviating oppressive systems [86].

It is evident from our findings that law enforcement and surveillance are having negative impacts on the lives of PWUD. A provincial legislative committee recently recommended reforming policing across the province, including replacing the federal RCMP with a provincial police force in order to increase accountability and improve police-community relations [87]. Others have called for interventions such as police education and training [23, 88, 89]. While these reform strategies are warranted, our findings also emphasize the need to address the harms caused by criminalization. Recently, the provincial government in British Columbia was granted a federal exemption to decriminalize the personal possession of some illicit drugs (e.g., opioids, cocaine, methamphetamine) for people 18 years of age or older in an effort to reduce stigma and save lives [90]. While we are supportive of policy interventions that address structural stigma, including the surveillance of PWUD, the province’s proposed accumulative threshold of 2.5 g for personal possession is too low and will negatively impact PWUD in rural communities [91], particularly those in remote and coastal communities who have to travel long distances to buy drugs in bulk in the city. Rural PWUD, thus, will not be adequately protected under this decriminalization model and will likely continue to experience surveillance. Further, it is not known if decriminalization will reduce the price of illicit drugs in British Columbia (this was not a result from Portugal’s decriminalization [92]). Therefore, additional measures such as providing PWUD with a government-funded regulated safer supply of drugs and evaluating this form of intervention may be warranted [93, 94]. This would not only likely reduce overdose risk, but also alleviate financial stressors, the need for PWUD to engage in criminalized activities to support their use, as well as the requirement to travel elsewhere and endure the surveillance and risk associated with acquiring illicit drugs in larger quantities in another city.

This study has its limitations. During data collection, there were pandemic-related restrictions that limited travel and in-person research activities and so we were unable to conduct ethnographic observation to further contextualize our study findings. Further, while we aimed to recruit a diversity of participants, our results may not be generalizable to all PWUD in the qathet region nor to those in other rural settings. In addition, although the ages of our participants ranged from 19 to 65, very few youth participated in this study and they may have unique experiences related to drug use in rural settings. Additionally, while there were some Indigenous participants, given the small sample size, variations in experience based on Indigeneity were not captured. Experiences of stigma and surveillance may be unique to Indigenous people in a rural setting given that they also face other systemic barriers and intersecting forms of discrimination (e.g., racism, colonialism) [17, 95]. Lastly, while some participants lived outside of Powell River (e.g., on islands or the outskirts of town), most participants were centrally located and within close proximity to harm reduction services and other forms of community supports compared to those living in underrepresented regions within qathet. These limitations highlight the need for further research in rural and coastal Canadian settings that is specific to underrepresented groups.

In conclusion, our study illustrates the unique experiences faced by people who use drugs in a rural and coastal Canadian setting. To our knowledge, this is the first study in Canada that examines the effects of social and structural contexts on the lives of PWUD in a rural and coastal community, and how the goldfish bowl effect created heightened social and structural surveillance leading to a variety of negative consequences. This study demonstrates a clear need for the implementation and evaluation of interventions to address the larger contextual drivers affecting PWUD in rural settings, including peer-led anti-stigma strategies, the decriminalization of PWUD, and a regulated supply of drugs in order to improve the well-being of PWUD in rural and coastal settings in Canada.

## Data Availability

The qualitative datasets for this study are not publicly available given the sensitive nature of the topic, as they contain confidential information that could compromise participant confidentiality and consent.

## Abbreviations

PWUD: People who use drugs
BC: British Columbia
RCMP: Royal Canadian Mounted Police
