# Correction: The “goldfish bowl”: a qualitative study of the effects of heightened surveillance on people who use drugs in a rural and coastal Canadian setting

**DOI:** 10.1186/s12954-024-01085-9

**Published:** 2024-09-11

**Authors:** Geoff Bardwell, Manal Mansoor, Ashley Van Zwietering, Ellery Cleveland, Dan Snell, Thomas Kerr

**Affiliations:** 1 School of Public Health Sciences, University of Waterloo, 200 University Avenue West, Waterloo, ON N2L 3G1 Canada; 2 British Columbia Centre on Substance Use, 400-1045 Howe Street, Vancouver, BC V6Z 2A9 Canada; 3 Department of Medicine, University of British Columbia, St. Paul’s Hospital, 608-1081 Burrard Street, Vancouver, BC V6Z 1Y6 Canada; 4 Qathet Community Action Team, 218-6975 Alberni Street, Powell River, BC V8A 2B8 Canada; 5 Lift Community Services of Qathet Society, 218-6975 Alberni Street, Powell River, BC V8A 2B8 Canada; 6 Substance Users Society Teaching Advocacy Instead of Neglect (SUSTAIN), 218-6975 Alberni Street, Powell River, BC V8A 2B8 Canada

**Correction to: Harm Reduction Journal (2022) 19:136 ** 10.1186/s12954-022-00725-2

Following publication of the original article [1], part of the first quotation in the Results section has been removed for legal reasons.

The original article has been corrected.
